# Components of professional satisfaction among novice nurses

**DOI:** 10.1186/s13584-023-00584-7

**Published:** 2023-11-21

**Authors:** Bella Savitsky, Rachel Shvartsur, Yifat Findling, Anat Ereli, Tova Hendel

**Affiliations:** Department of Nursing, School of Health Sciences, Ashkelon Academic College, Yitzhak Ben Tzvi 12, 78211 Ashkelon, Israel

**Keywords:** Nursing baccalaureate, Nursing education, Job satisfaction, Professional development

## Abstract

**Background:**

In Israel there are only 6.53 nurses per 1000 citizens, compared to 8.8 nurses per 1000 citizens in the OECD countries. The nursing shortage is even more severe in peripheral areas, especially in southern Israel. Nurses` professional satisfaction is crucial for preserving the nursing workforce. This study aimed to assess job satisfaction among novice nurses and identify components of professional satisfaction.

**Methods:**

Cross-sectional study of 216 novice nurses who graduated in 2018–2022 and were approached ten months after graduation. Job satisfaction components were constructed using factor analysis.

**Results:**

Professional satisfaction was based mainly on the intrinsic characteristics of the occupation related to personal accomplishment and organizational culture. In a multivariable model, a one-point increase in mean satisfaction with the training period during studies in the nursing department was associated with a more than a three-fold elevation in the odds for high and very high professional satisfaction (OR 3.0, 95% CI 1.7–5.1). Odds for high and very high professional satisfaction were more than four-fold and two-fold higher among graduates who rated their level of control over work schedule as high and medium vs. low (OR 4.2, 95% CI 1.0–16.7 and OR 2.8, 95% CI 1.2–6.3, respectively). Work-life balance without disturbance to daily life by work was found significantly associated with higher odds for high and very high satisfaction. Nurses who plan to continue professional development, i.e., an advanced professional course or Master’s degree, had significantly higher mean professional satisfaction scales than others (4.2 vs. 3.7, *p* = .009 and 4.2 vs. 3.9, *p* < .001, respectively).

**Conclusion:**

The most important components of professional satisfaction among novice nurses are self-accomplishment, which was built from work-related challenges, interest and variety of tasks, personal growth and development, and the possibility of contributing to patients` care and organizational culture, which was built from relationships with co-workers. Persons who manage nurses should cultivate an atmosphere of support and guidance, provide new nurses with interesting work tasks, and increase their ability to control their work schedule. Young nurses should be encouraged to continue their professional and academic education.

## Introduction

COVID-19 has brought healthcare workers in general, and nurses in particular, into the limelight as never before. The worldwide nursing shortage is now widely acknowledged as a serious problem that is at or near crisis levels [1]. According to a survey conducted by the US National Council of State Boards of Nursing (NCSBN), nearly 100,000 registered nurses left the field of nursing during the COVID-19 pandemic due to stress, burnout, and retirement, and almost 800,000 (28.7% of all US nurses) intend to follow them by 2027 [2]. In Israel, the ratio of licensed nurses (up to the age of 67) per 1000 citizens stands at 6.53 nurses per 1000 citizens, which is lower than the OECD countries' average of 8.8 nurses [3, 4]. The nursing shortage is even more severe in peripheral areas, especially in southern Israel. According to the Ministry of Health, the number of registered nurses per 1000 citizens is 7.9 and 6.2 in Haifa and Tel Aviv districts, compared to 4 per 1000 citizens in the South [4]. Moreover, only 84–85% of Israeli licensed nurses actively work in the profession [5, 6], and more than half of the employed registered nurses are aged 45 or older [6, 7]. Although the ratio of licensed nurses per 1000 citizens has gradually increased over the last two decades, the upward trend is notably sluggish, and the annual influx of new nurses into the healthcare system fails to keep pace with population growth [8]. The projected shortfall of nurses in Israel is expected to reach 38,000 by 2030 [8]. A low number of nurses signals a health system operating below its optimal capacity, falling short of meeting the demand for adequate and necessary care and treatment for every patient, both in hospitals and within the community. Hence, recruiting young individuals into the profession is crucial.

The initial six to twelve months of practice, known as the transition period, is the most critical and vulnerable phase for newly graduated nurses to decide whether to stay in or exit the nursing profession [9, 10]. Studies suggest that during the first year in the profession, newly graduated nurses often encounter high-stress work environments and may exhibit a tendency towards higher turnover [2, 11, 12]. Novice nurses with underdeveloped social belongingness, insufficient confidence in skills, and limited nursing experience are susceptible to workplace bullying by more senior nurses, with a reported 30% prevalence during their first year [13]. Novice nurses report fears of knowledge gaps and feeling overwhelmed by high workloads, staff shortages, lack of experience, and high expectations of colleagues [12, 14]. This may potentially jeopardize patient safety [15] and decrease professional satisfaction and motivation to stay in the profession [12, 16]. Concrete and up-to-date information on turnover rates, specifically for novice nurses, is scarce. The report published in 2022 by Nursing Solutions (NSI) found that 31% of novice nurses leave their jobs within the first year of employment [17]. In New Zealand, a third of new graduates leave the nursing profession with no intention to return [18]. A quantitative systematic review of 34 studies found that among new graduate nurses, the intention to resign was predicted by factors related to stress and general and work-related well-being [16].

Except for leaving the profession, low satisfaction can lead to employees leaving their workplace or the organization. According to most turnover models, it serves as a key factor influencing an employee’s decision to stay with or leave an organization [19, 20]. Additionally, extensive empirical evidence consistently supports the association between job satisfaction and turnover [21–29]. Therefore, creating supportive work environments that enhance job satisfaction among new graduate nurses could be instrumental in retaining them [10, 14]. Novice nurses possess distinct characteristics, and it's possible that the factors influencing their professional satisfaction may vary from those of experienced nurses. While there is substantial evidence linking job satisfaction with nurse turnover generally, the evidence among new graduates is limited [25].

In addition to its impact on the quality of care and the risk of nurse turnover, professional satisfaction is essential for nurses' overall well-being and health [30–32]. Low professional satisfaction may lead to anxiety and stress [31]. Conversely, high job satisfaction has been linked to improvements in both physical and mental health, as well as increased levels of happiness and self-esteem. These positive factors collectively contribute to enhanced organizational performance [30, 32].

Our study aimed to assess the job satisfaction and postgraduate job transition period among Bachelor Science of Nursing (BSN) graduates ten months after graduation to determine the components of professional satisfaction and identify specific strategies to improve the professional satisfaction of novice nurses.

## Methods

### Study design

This cross-sectional study included graduates of a four-year BA program of five classes (2018–2022) who have graduated since the Department of Nursing at the Ashkelon Academic College established the baccalaureate program in 2014. Ten months after graduation, we contacted all 252 graduates via e-mail and requested them to complete a questionnaire created using Google Forms. Thus, graduates who started their nursing program in 2014 and finished in 2018 were approached in 2019, graduates who started in 2015 and finished in 2019 were approached in 2020, etc. Out of 252 graduates who were approached, 234 filled out the survey, resulting in a response rate of 93%. This response rate was achieved using reminders by e-mail by different instructors. We chose this approach since we considered that sometimes students had closer relationships with specific instructors.

### Study population

Eighteen former students who responded and reported that after graduation they did not start working as nurses were excluded from the study. Thus, the study population comprises 216 novice nurses.

### Ethical consideration

All participants signed an informed consent to participate. The study received approval from the Ethical Board of the Department of Nursing at the Ashkelon Academic College (Approval no. 11-11-2020).

Strengthening the Reporting of Observational Studies in Epidemiology (STROBE) statement chosen for reporting study results.

### Demographic variables

Demographic variables in the survey instrument included:

Age (used as a continuous variable); Sex (female; male); Family status (married/in relationship; single/divorced); Parental status (does not have children; has children); Country of birth (Israel; other countries); Ethnicity group (Jew; Arab and Bedouin); Level of religiosity (secular [nonobservant]); traditional [observes some religious commandments]; religious [observes all religious commandments]).

### Satisfaction with the nursing program in the faculty of nursing

The survey included 20 items for assessment of satisfaction with theoretical courses and clinical practice in 10 nursing fields of study. Items were rated on a 5-point Likert-type scale (1 = to a very small degree to 5 = to a very high degree). Mean satisfaction with nursing programs in the faculty of nursing (continuous variable) was calculated as a sum of 20 items, divided by 20. This tool was adapted from the study that investigated satisfaction with the teaching program among young physiotherapists [33]. We tested the reliability of this tool in the current study (Cronbach's Alpha method = 0.9).

### Professional variables

We collected information on: Place of work (hospital; community); Region of work (south; center; north; Jerusalem and environs); Form of Employment (full-time job; part-time job); Level of control over work schedule (low; medium; high).

Frequency of work disturbances to everyday life (never; sometimes; always).

Participants were asked about their career planning, i.e., whether they are planning to continue their professional or academic education. Based on their answer, two dichotomous variables were created. Advanced course (planning to participate or already participating vs. not in the plan) and Master’s degree (planning to study or already studying vs. not in the plan).

### Professional satisfaction

The final version of the questionnaire included 26 items related to professional satisfaction:

Twenty-two items from the Minnesota Satisfaction Questionnaire (MSQ) (short version translated to Hebrew) [33]. The reliability of the MSQ questionnaire in Israeli studies using Cronbach`s Alpha method was 0.95 [34] and 0.89 [35]. Four additional items were included in the final version following the request of three senior nurses working in a general hospital.

Items were rated on a 5-point Likert-type scale (1 = to a very small degree to 5 = to a very high degree). Mean Professional Satisfaction (continuous variable) was calculated as a sum of items constructing a professional satisfaction questionnaire, divided by the number of items with responses (as not all participants ranked all 26 items). The reliability of this tool in this study was high (Cronbach's Alpha method = 0.94).

Two categories of professional satisfaction were created based on mean professional satisfaction: a score of 4 and above was called "high and very high professional satisfaction", score < 3.9 was defined as low and medium professional satisfaction.

### Statistical analysis

The association between the mean professional satisfaction score and demographic/professional characteristics was assessed using the Mann–Whitney non-parametric test (if two samples were compared) or the Kruskal–Wallis non-parametric test (if more than two sub-samples were compared). The Pearson correlation coefficient was used to assess the correlation between the mean professional satisfaction score and satisfaction with the current workplace. We also performed multivariable analysis using backward logistic regression. The dependent variable was the probability for high and very high professional satisfaction. The independent variables included sex, mean satisfaction with nursing program in the faculty of nursing, place of work (hospital vs. community), level of control over work schedule, and frequency of disturbance by work. Before including independent variables in the multivariable analysis, the correlation between the variables was checked with Kendall's Tau coefficient to prevent multicollinearity.

Professional satisfaction components were constructed using factor analysis with a Varimax rotation and an unrestricted number of factors. Variables with factor loadings ≥ 0.5 were considered contributing variables to a given factor.

For all analyses performed, a value of p < 0.05 was considered statistically significant. Analyses were carried out with the SPSS version 25.0 statistical package (SPSS, Inc, Chicago, IL).

## Results

### Demographic characteristics of the study population

The mean age of the 216 novice nurses in the study population was 27.8 (SD = 3.3 years); 88% were women. As presented in Table 1, no significant differences were detected among the nurses from the different graduating classes in any of the demographic parameters.

**Table 1:** Demographic and professional characteristics of the study population by graduating class

Demographic and professional characteristics	Graduating class					
	2014–2018	2015–2019	2016–2020	2017–2021	2018–2022	Total
	n = 30	n = 45	n = 43	n = 50	n = 48	*n* = 216
**Age (years)** mean (SD)	28.0 (3.6)	28.8 (3.9)	27.8 (3.0)	27.5 (3.6)	27.3 (2.0)	27.8 (3.3)
**Sex (%)**						
Female	90.0	93.3	86.0	88.0	83.3	88.0
Male	10.0	6.7	14.0	12.0	16.7	12.0
**Married or living with a partner (%)**	70.0	57.8	67.4	64.0	79.2	67.1
**Has children (%)**	46.7	33.3	39.5	46.0	22.9	37.0
**Birth country (%)**						
Israel	66.7	75.6	79.1	82.0	75.0	76.4
Other^a^	33.3	24.4	20.9	18.0	25.0	23.6
**Ethnicity "Jew" (%)**	96.7	95.3	90.5	98.0	95.8	98.1
**Level of religiosity (%)**						
Secular	50.0	57.8	47.6	46.9	60.4	52.8
Traditional	20.0	15.6	31.0	20.4	14.6	20.1
Religious	30.0	26.7	21.4	32.7	25.0	27.1
**Place of work (%)**						
Hospital	90.0	82.2	88.4	80.0	73.9	82.6
Community	10.0	17.8	11.6	20.0	26.1	17.4
Form of employment (%)*						
Full-time job	–	15.6	67.4	64.0	70.8	47.2
Part-time job	100	84.4	32.6	36.0	29.2	52.8

*SD* - standard deviation

^a^93% of immigrants came from the Former Soviet Union (FSU)

*p < .05

### Professional transition

The median time of employment seeking was seven days (IQR 0–30), while 53.7% of graduates found a job within a week. For 17.4% of graduates, their first job was in the community (mainly HMO community clinics). Among the others (82.6%), the first workplace was a hospital. The most frequent departments were the emergency department (n = 37), the internal medicine department (n = 28), and the obstetrics and gynecology department (n = 22). Most graduates (n = 137, 63.4%) reported working in the workplace that they defined as their priority. As far as location is concerned, 68.5% of graduates were working in the south of Israel, 26.9% in the center, 1.9% in the Jerusalem area, and 2.3% in the north of the country.

Except for the first two classes, where the rate of novice nurses who worked full time was low, among the others, more than 60% reported working full time.

### Professional satisfaction

The mean professional satisfaction score in the study population was 4.2 (SD = 0.6; median 4.2, IQR [3.9–4.6]). Most graduates reported high and very high satisfaction with the profession (n = 142, 65.7%) and current workplace (n = 168, 77.8%). The mean professional satisfaction score and satisfaction with the current workplace were highly correlated (r = 0.7, *p* < 0.0001).

**Fig. 1:**
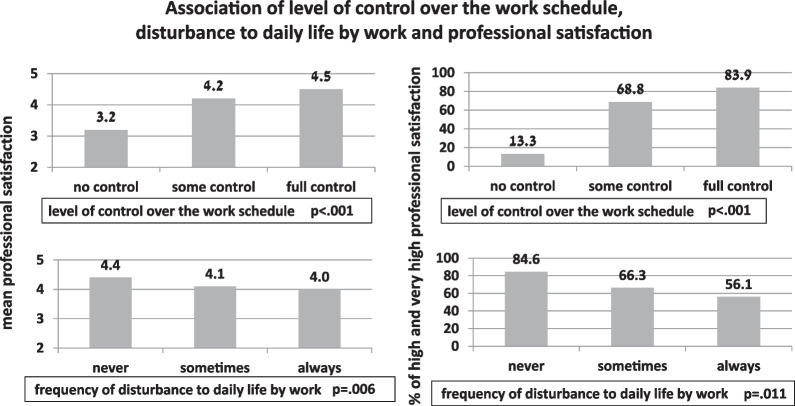
Association of level of control over the work schedule, disturbance to daily life by work, and professional satisfaction

None of the demographic characteristics were significantly associated with professional satisfaction (data is not presented). Professional characteristics such as working in the community or hospital, type of department, and being accepted to their priority workplace were not significantly associated with professional satisfaction. A higher proportion of those novice nurses who work a partial-time job reported high and very high professional satisfaction (71.1%) than those, who reported working a full or part-time job (59.8%); this difference had a borderline significance (p = 0.06). The level of control over the work schedule and the level of disturbance created by work to daily life were significantly associated with professional satisfaction (Fig. 1), while higher mean professional satisfaction score (and higher proportion of those, who reported high and very high professional satisfaction) were found among those novice nurses who reported low level of disturbance and higher control over the work schedule.

### Professional Satisfaction (a multivariable analysis) (Table 2)

**Table 2:** Odds for high and very high professional satisfaction: Results of the Multivariable Logistic regression model

Characteristics	Odds ratio (OR)	95% CI	p value*
**Sex** (female versus male)	1.3	0.5–3.7	0.56
**Place of work** (hospital versus community)	2.4	1.0–5.8	0.05
**Mean satisfaction with training during the study in the nursing department**	3.0	1.7–5.1	< 0.001
**Level of control over work schedule**			
Medium versus low	2.8	1.2–6.3	0.016
High versus low	4.2	1.0–16.7	0.045
**Frequency of disturbance to daily life by work**			
Sometimes versus always	1.7	0.8–3.7	0.19
Never versus always	3.3	1.0–10.9	0.05

*p = 0.05 is considered as a value with borderline significance, and *p* < 0.05 is considered statistically significant

An increase in mean satisfaction with the nursing education program by one point was associated with a three-fold elevation in the odds for high and very high professional satisfaction (OR 3.0, 95% CI 1.7–5.1). Novice nurses who work in the hospital had more than twice the odds for high and very high professional satisfaction in comparison with those, whose first workplace was in the community (OR 2.4, 95% CI 1.0–5.8). Odds for high and very high professional satisfaction were more than four-fold and three-fold higher among nurses who rated their level of control over work schedule as high and medium (vs. low) (OR 4.2, 95% CI1.0–16.7) and OR 2.8, 95% CI 1.2–6.3, respectively). Never being disturbed by the work was associated with more than three-fold odds of having high and very high professional satisfaction as compared to always (OR 3.3, 95% CI 1.0–10.9).

The model explained 27% of the variance in the probability of high and very high professional satisfaction.

### Components of professional satisfaction

Four factors together explained 65.4% of the variance in professional satisfaction. The components of each factor are depicted in Table 3. The first factor, referred to as *self-accomplishment,* explained a very significant scope (45.4%) of variance. This factor was related to feelings of worthwhile accomplishment, a challenge at work, the extent of diversity and interest in the professional duties, the use of skills, personal growth and development, contribution to the patient’s care, prospect for promotion, and the extent of independency in the job.

**Table 3:** Loading coefficients of factors obtained by factor analysis

Factor I 45.4%	Factor II 9.5%	Factor III 6.0%	Factor IV 4.5%
Self-accomplishment	Organizational culture	Work conditions and reward	Managerial support
The feeling of worthwhile accomplishment I get from my work 0.85	The relationships with colleagues (other nurses in my workplace) 0.79	The number of staff members who work with me 0.80	The degree to which my supervisor relates to the staff 0.83
The amount of challenge in my job 0.83	The amount of support I receive from my coworkers 0.78	The amount of time available to finish everything I have to do 0.79	The degree to which I see my supervisor serve as a role model 0.79
The extent to which my job is varied and interesting 0.80	The degree to which I feel part of a nursing team 0.77	The amount of time spent on administration 0.76	The amount of support and consideration of my needs I receive from my supervisor 0.76
The extent to which I can use my skills 0.79	The quality of the guidance I get at work 0.68	The degree to which my salary fits my efforts at work 0.53	
The amount of personal growth and development I get from my work 0.78	The relationships with multi-professional team at my workplace 0.62		
The contribution to patients' care 0.72			
My prospects for promotion 0.57			
To the extent that I can make independent decisions in my work 0.55			

The second factor, referred to as* organizational culture*, explained 9.5% of the variance. This factor increased with an increase in satisfaction with relationships with colleagues (other nurses), the amount of support from coworkers, the degree to which a nurse feels part of a nursing team, the quality of the guidance at work from other nurses, and relationships with multi-professional staff.

The third factor, referred to as *work conditions* and *reward*, explained 6% of the variance. This factor was related to satisfaction from the workload (number of staff members), the scope of duties and the degree to which salary fits efforts at work.

The fourth factor, referred to as *managerial support*, explained 4.5% of the variance. As the satisfaction with the relationship with the head nurse (the degree to which the supervisor relates to the staff, serves as a role model, and supplies enough support) increased, so did this factor.

### Professional satisfaction and professional plans

Graduates who plan to continue professional development by taking an advanced professional course have significantly higher mean professional satisfaction than those who are not planning to continue their professional education (4.2 vs. 3.7, *p* = 0.009). Those who reported having plans to continue their academic education to the master's degree had higher mean occupational satisfaction (4.2) than those who do not (3.9) (*p* < 0.001).

Novice nurses who reported high or very high professional satisfaction reported a significantly higher willingness to recommend a nursing career to friends and family members (4.3 vs. 3.7, *p* < 0.001).

## Discussion

The current research aimed to study the professional satisfaction of novice nurses during the first year after graduation. High professional satisfaction was found, with self-accomplishment and organizational culture identified as the most significant components of satisfaction among novice nurses. The mean professional satisfaction in this study (4.2) is higher than those found in previous studies among senior nurses. In the Israeli nursing study conducted by Savitsky et al. in 2020, nurses with an average professional experience of 13 years reported a mean professional satisfaction of 3.6 [36]. It's worth noting that this study was conducted during challenging pandemic circumstances. In another Israeli study conducted in 2014, the mean professional satisfaction was 3.9 [37]. Similar results have been reported in other countries. In 2006, a study conducted among Chinese nurses [38] reported a mean professional satisfaction of 3.3, while studies conducted in Finland [39] and Portugal [40] found mean professional satisfaction of 3.6 and 3.7, respectively. All these studies were conducted under regular (non-pandemic) conditions among nurses with some seniority in the profession. Ma et al., reported higher levels of job satisfaction among inexperienced nurses (0–2 years) as compared to experienced nurses (over two years) [41].

We found that satisfaction with the program in the nursing department was associated with higher professional satisfaction significantly and independently from other factors. Similar results were found in 2016 in an Australian study, where satisfaction with nursing education was a significant predictor of job satisfaction [42]. Likewise, research conducted among recently graduated physical therapists in Israel discovered that job satisfaction was predicted by the extent to which respondents felt satisfied with their study program [33]. We believe that self-confidence serves as the intermediary factor in the association between satisfaction with the study process and professional satisfaction [43]. Self-confidence is defined as the belief of individuals in their own abilities to achieve goals and perform tasks and practices effectively and efficiently [44]. The professional self-confidence of nurses develops when nursing students gain theoretical knowledge and critical thinking skills and further consolidate their evidence-based learning in clinical and academic environments [45]. The transition from a former student to a novice nurse is challenging [46]. Various challenges are waiting for young nurses, and those who are better prepared will feel more confident and will achieve higher professional satisfaction.

The main component of professional satisfaction, *self-accomplishment*, was built from work-related challenges, interest and variety of tasks, personal growth and development, and the possibility of contributing to patients` care. The workforce of young nurses is highly motivated; thus, preserving their professional inspiration and preventing burnout is crucial.

An additional component of professional satisfaction is *organizational culture*, containing support and relations with other nurses and multi-professional staff. A large body of evidence demonstrates the importance of interpersonal relationships to nurses’ job satisfaction [47–52]. Adams and Bond found that the most important contributors to nurses’ job satisfaction were the degree of cohesion of the ward nursing team, the degree of collaboration with medical staff, and the perception of staff organization [48]. Another study found that good collegial relationship with co-workers is one of two factors that are dominant in nurses’ understanding of job satisfaction, along with the perceived ability to deliver good patient care [52]. Co-workers` support was even more crucial during the pandemic: a survey among 2500 nurses in New York City who worked during the first wave of the pandemic revealed that 75% of nurses indicated that co-worker support was beneficial to their mental well-being [53]. A study from the Netherlands in which diary entries of novice nurses with less than a year seniority in the profession were analyzed discovered that lack of support from colleagues significantly influenced job commitment [54].

Another component of professional satisfaction related to the *reward* and *work conditions* was significantly less important to novice nurses as compared to self-accomplishment and organizational culture. These findings support Frederick Herzberg’s two-factor theory [55] arguing that intrinsic job characteristics related to personal growth, accomplishments, professional challenges, diversity, and interest (not extrinsic factors such as work conditions and economic reward) contribute to professional satisfaction. On the other hand, the study shows that lower control over the work schedule and higher level of disturbance to daily life outside of work are significantly associated with lower professional satisfaction. Accordingly, high work-family conflict was associated with a lower quality of nursing care in the study conducted among nurses working in the COVID-19 ward [56]. Work-to-family conflicts negatively affect family outcomes and employee physical and mental health [57]. Conflicts between work and family result in lower professional satisfaction [49, 57, 58] and more intentions to quit [59, 60].

The last component of *managerial support* highlights the importance of the relationship with the head nurse. Given the importance of managerial support, nurse managers need to possess the necessary competencies to enact supportive role behaviors [61]. It is important for nurse managers to have access to human resource management programs, which should ideally be made mandatory. These programs enable nurse managers to develop the necessary competencies to exhibit supportive role behaviors and foster a work environment characterized by cooperation and support within their department. Nurses’ professional satisfaction is one of the most important factors in determining individuals’ intention to stay or leave a healthcare organization [62–65] or the profession [62, 63]. Laschinger (2012) studied job and career satisfaction and turnover intentions of newly graduated nurses in their first and second year of experience. In this study, occupational dissatisfaction was one of the significant predictors of the intention to leave the nursing profession [62]. In line with these studies, we found that novice nurses with higher professional satisfaction expressed willingness to take an advanced professional course. A study previously conducted in Israel found that nurses' willingness to stay in the profession was strongly predicted by the existence of advanced practice courses in their curriculum vitae [66].

Finally, highly satisfied novice nurses report willingness to recommend their friends and family members to choose nursing as a profession. Thus, promoting high professional satisfaction is important not only for the nurses' functioning and well-being but also for the thriving of the nursing profession.

### Policy implications and recommendations

This research project revealed the main components of novice nurses` professional satisfaction and may contribute to preserving nurses in the profession by increasing their professional satisfaction. The level of professional satisfaction appears to be related not only to the degree to which novice nurses find their jobs challenging but also to work-family conflicts. Therefore, it is important both to provide new nurses with interesting work tasks and to increase their ability to control their work schedule.

Those who manage nurses, such as head nurses, should actively try to develop meaningful relationships at work with novice nurses and promote relationships of communication and cooperation between novice and senior nurses. The novice nurses should feel that they are being supported and guided in their careers by more senior staff. Good communication with novice nurses and a culture of collaboration should contribute to their professional satisfaction.

Young nurses should have an opportunity and receive economic and conceptual support to continue their professional and academic education, i.e., taking advanced courses or pursuing Master’s degrees. Advanced education contributes to higher professional satisfaction and connects nurses to the profession. Since the satisfaction of novice nurses with studying and learning during the bachelor program in the nursing department is associated with higher professional satisfaction, the quality of the teaching should be assessed frequently with an eye to continuous improvement.

The study revealed that our graduates found their first place of work very quickly. Notably, 68.5% of novice nurses in our sample are employed in the southern region of Israel. These findings highlight the crucial role of nursing education facilities in peripheral areas, where workforce shortages are particularly acute.

### Study strength

The current study was conducted with the entire population of graduates and not with a sample, with a very high response rate, excluding the possibility of selection bias. The study population was representative in relation to the Israeli population of graduates, considering the distribution of sex, ethnicity, level of religiosity, and family status. Professional satisfaction in this study was assessed using a research tool with high content validity, including extrinsic and intrinsic aspects of professional satisfaction. The usage of Factor Analysis made it possible to define the main components of professional satisfaction among novice nurses.

### Study limitations

Because of the cross-sectional design of the study, one cannot draw firm conclusions about the causality of the associations found in the study.

Another study's limitation lies in its quantitative approach. A qualitative study conducted prior to the quantitative one can potentially obtain a richer set of factors than might emerge from survey data, but it faces challenges in achieving a "representative sample" due to the limited number of participants providing extensive information.

## Conclusions

High professional satisfaction is crucial for nurses’ psychological well-being and physical health. Satisfaction with the occupation prevents turnover and readiness to leave the profession. In this study, novice nurses were found to be highly motivated. However, there is still work to be done to improve new graduates' work environments. Retaining new graduate nurses is essential for addressing the nursing shortage and for sustaining the future of the profession. Addressing the factors associated with high professional satisfaction should be a high priority for organizations that employ novice nurses.

## Data Availability

The analyzed dataset and the syntax file are available from the corresponding author upon reasonable request.
